# Access to fluorochemicals directly from fluorspar

**DOI:** 10.1038/s42004-023-00987-2

**Published:** 2023-08-24

**Authors:** Huijuan Guo

**Affiliations:** Communications Chemistry, https://www.nature.com/commschem

## Abstract

Fluorochemicals have a wide range of applications in industry, but accessing these relies on the energy intensive conversion of acid-grade fluorspar (CaF_2_) to toxic hydrogen fluoride (HF) gas, which is in turn used for the downstream production of fluorochemicals via multistep processes. Now, directly treating acid-grade fluorspar with dipotassium hydrogen phosphate (K_2_HPO_4_) under mechanochemical conditions affords a fluorinating reagent for direct S–F and C(sp^3^/sp^2^)–F bond construction, bypassing the need for HF production.

Simple inorganic fluoride salts are often insoluble in the solvents that dissolve their reaction partners. While bio-inspired urea organocatalysts have been developed to make potassium fluoride (KF) and cesium fluoride (CsF) soluble through hydrogen bonding, enabling enantioselective fluorinations with these alkali-metal fluorides^1^, using calcium difluoride (fluorspar; CaF_2_) as a fluorinating reagent remains very challenging owing to its high insolubility. So far, only limited fluorochemicals such as LiPF_6_, PF_5_, POF_3_ and Ca(SO_3_F)_2_ are produced using inorganic CaF_2_, and under extremely harsh conditions^2–4^.

Now, a team led by Véronique Gouverneur from Oxford University in the UK shows that potassium phosphate salts can be used to activate CaF_2_ in the solid-state by mechanochemistry, providing direct access to fluorinated products while bypassing the need for toxic HF production (10.1126/science.adi1557)^5^. “The vision in my laboratory is to rejuvenate fluorine chemistry with global challenges in mind. The technology we have invented is a first step in this direction and sets the stage to create a new circular fluorochemicals economy”, says Gouverneur.

Mechanochemistry is able to initiate reactions independently of solubility, and aid solid-state diffusion kinetics. Here, the team took inspiration from calcium phosphate (bio)mineralization, such as in the formation of bones and teeth, with a view to using the formation of a calcium phosphate by-product as a driving force for fluorination. Pleasingly, treating industry standard acid-grade fluorspar with K_2_HPO_4_ was found to be an effective protocol for CaF_2_ activation. The resultant powdered product, Fluoromix, was found to be suitable for forging S−F and C(sp^3^/sp^2^)–F bonds in solution, generating over 50 fluorochemicals in high yield (Fig. 1).

**Fig. 1:**
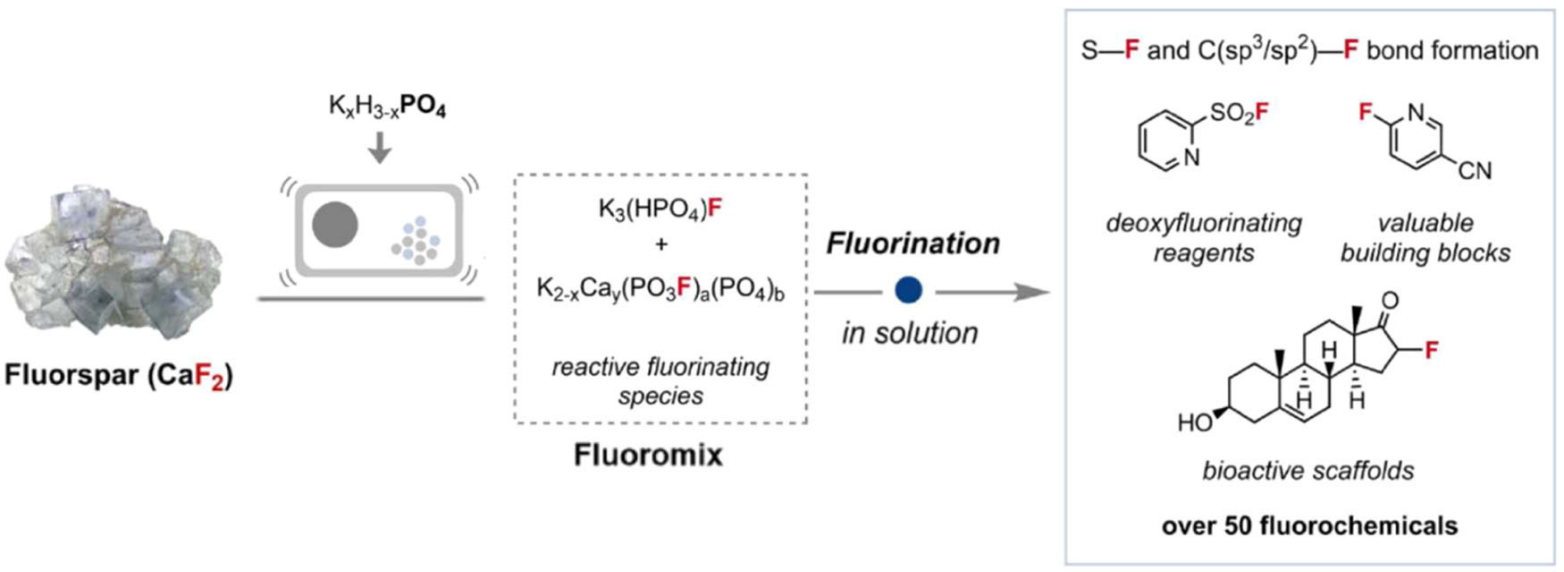
Synthesis of fluorochemicals from fluorspar (CaF_2_). Mechanochemical generation of Fluoromix from acid-grade fluorspar, and application to produce various fluorochemicals. Adapted with permission from Science https://www.science.org/doi/10.1126/science.adi1557. Copyright (2023) AAAS.

“One of the biggest challenges was deciphering the underlying processes in the mechanochemical activation of CaF_2_”, comments first author Calum Patel. “We initially hypothesized that the species formed could be potassium fluoride (KF) and a calcium phosphate by-product (or derivative thereof), but this was more complex than anticipated.” Powder X-ray diffraction was used to monitor the consumption of CaF_2_ by the potassium phosphate salt, and the reaction between KF and K_2_HPO_4_ was key to determining the composition of Fluoromix. Indeed, the product of this reaction, K_3_HPO_4_F, was observed in Fluoromix, and when ball milled with calcium hydrogen phosphate (CaHPO_4_), a new product (K_*2-x*_Ca_*y*_(PO_3_F)_*a*_(PO_4_)_*b*_) formed, which was also found to be present in Fluoromix. “Prof. Michael Hayward was instrumental in determining the structures of these new salts, both of which possess fluorinating abilities”, says Patel.

“We hope that this technology will enable us to access a wider fluorochemical space, specifically critically needed and non-persistent fluorochemicals. Ultimately, we hope this work will inspire scientists across the globe to provide disruptive solutions to chemical challenges.” concludes Patel.
